# Pancreatic Transcription Factors Containing Protein Transduction Domains Drive Mouse Embryonic Stem Cells towards Endocrine Pancreas

**DOI:** 10.1371/journal.pone.0036481

**Published:** 2012-05-01

**Authors:** Maria João Lima, Hilary M. Docherty, Yuanxiao Chen, Ludovic Vallier, Kevin Docherty

**Affiliations:** 1 School of Medical Sciences, University of Aberdeen, Institute of Medical Sciences, Foresterhill, Aberdeen, United Kingdom; 2 The Anne McLaren Laboratory for Regenerative Medicine, Cambridge, United Kingdom; Baylor College of Medicine, United States of America

## Abstract

Protein transduction domains (PTDs), such as the HIV1-TAT peptide, have been previously used to promote the uptake of proteins into a range of cell types, including stem cells. Here we generated pancreatic transcription factors containing PTD sequences and administered these to endoderm enriched mouse embryonic stem (ES) cells under conditions that were designed to mimic the pattern of expression of these factors in the developing pancreas. The ES cells were first cultured as embryoid bodies and treated with Activin A and Bone morphogenetic protein 4 (BMP4) to promote formation of definitive endoderm. Cells were subsequently plated as a monolayer and treated with different combinations of the modified recombinant transcription factors Pdx1 and MafA. The results demonstrate that each transcription factor was efficiently taken up by the cells, where they were localized in the nuclei. RT-qPCR was used to measure the expression levels of pancreatic markers. After the addition of Pdx1 alone for a period of five days, followed by the combination of Pdx1 and TAT-MafA in a second phase, up-regulation of insulin 1, insulin 2, Pdx1, Glut2, Pax4 and Nkx6.1 was observed. As assessed by immunocytochemistry, double positive insulin and Pdx1 cells were detected in the differentiated cultures. Although the pattern of pancreatic markers expression in these cultures was comparable to that of a mouse transformed β-cell line (MIN-6) and human islets, the expression levels of insulin observed in the differentiated ES cell cultures were several orders of magnitude lower. This suggests that, although PTD-TFs may prove useful in studying the role of exogenous TFs in the differentiation of ES cells towards islets and other pancreatic lineages, the amount of insulin generated is well below that required for therapeutically useful cells.

## Introduction

Type 1 diabetes is an autoimmune disease, in which the β-cells in the islets of Langerhans are specifically destroyed. The disease is currently treated with multiple daily injections of insulin, however it is very difficult using exogenous insulin to prevent hypoglycaemic episodes and the debilitating late complications of the disease. Islet transplantation may represent a potential form of treatment [1], but the poor availability of donor tissue prevents its widespread use. For this reason alternative sources of β-cells from human pluripotent cells has been sought [2].

Most of the protocols that have been established to drive pluripotent cells towards the β-cell lineage involve inducing the formation of a definitive endoderm (DE) enriched population by using Activin A [3], [4], a member of the TGFβ family of growth factors. From there the cells are directed down a differentiation pathway that mimics the events that occur in the developing mouse [5]. The idea is to recapitulate the pattern of expression of key transcription factors, including Pdx1, Ngn3, NeuroD, Nkx6.1, Pax4, and MafA, that define the β-cell lineage [6]. This approach has been validated by controlling the temporal expression of an exogenous Pdx1 gene in ES cells that have been stably transfected with a tetracycline responsive Pdx1 DNA construct [6]. This ability to fine tune the activity of key transcription factors in a dose and time dependent manner may overcome some of the challenges in generating functional β-cells *in vitro*.

Most cells are designed to prevent non-specific uptake of proteins. However, certain viruses have circumvented this by generating proteins that contain protein transduction domains (PTDs) [7]. Thus the PTD of the HIV1-TAT protein has been exploited in cell engineering because of its effectiveness and small size [8], [9], and TAT-fused proteins have been shown to be efficiently taken up by a variety of cells, including ES cells [10]–[12].

In the present study we used PTD-mediated uptake of Pdx1 and MafA into DE-enriched mouse ES cells. These transcription factors were added in a manner that mimicked the temporal pattern of expression in the mouse embryo. The results showed that insulin expressing β-like cells could be generated. However, although a similar expression pattern of pancreatic markers was observed for the differentiated ES cells, insulin expression levels were very much lower when compared to those of the MIN-6 β-cell line or human islets, suggesting that PTD-TFs may have a limited role in protocols designed at generating ES-derived islet cells for therapeutic purposes.

## Methods

### Cell Culture

HeLA cells (ATCC, Teddington, UK) were cultured in Dulbecco's Modified Medium (DMEM) containing 15% foetal calf serum (FCS). The mouse CGR8 ES cell line [13] was cultured on gelatin coated tissue culture dishes in medium composed of KO- DMEM supplemented with 15% KO Serum Replacement, 1× nonessential amino acids, 1× L-glutamine, 1% β-mercaptoethanol (all from Invitrogen, Paisley, UK) and leukemia inhibitory factor (LIF). CGR8 differentiation was done in chemically defined medium (CDM) [14] composed of Iscove's Modified Dulbecco's Medium/F12, 1× chemically defined lipids (both from Invitrogen), 7 µg/ml insulin, 5 mg/ml BSA, 450 µM monothioglycerol (all from Sigma, Dorset, UK), 15 µg/ml transferrin (Roche Diagnostics, West Sussex, England) in suspension cultures for embryoid bodies (EBs) formation. For DE formation cells were cultured as EBs in CDM supplemented with 100 ng/ml Activin A and 10 ng/ml BMP-4 for 2 days and with Activin A alone for another 4 days [6]. For pancreatic differentiation EBs were plated in cell culture plates covered with Matrigel™. Cells were then cultured for 21 days in KO-DMEM in the absence or presence of 1 µM Pdx1 and/or TAT-MafA. MIN6 cells (ATCC, Teddington, UK) were cultured in DMEM containing 25 mM glucose supplemented with 15% FCS (both from Gibco) and 70 µM β-Mercaptoethanol (Sigma).

### Human Islets

Human islets of Langerhans were obtained with appropriate ethical approval in place. Upon arrival the fresh islet tissue was stored in Trizol® reagent (Invitrogen) for subsequent RNA extraction and RT-qPCR analysis.

### Vector construction and protein purification

The His6-Pdx1 construct was generated by inserting the coding sequence of human Pdx1 into a pQE-31 expression vector (Qiagen, West Sussex, UK). The His6-TAT-MafA and the His6-TAT-Pdx1 constructs were generated by inserting mouse MafA and human Pdx1 coding sequences into a pET 28b TAT vector. These constructs were used to transform the competent *E. coli* strain BL21 (DE3) (Invitrogen). Transformed BL21 (DE3) cultures expressing the protein of interest were used to inoculate 2 L of LB medium supplemented with kanamycin and ampicillin and grown for 1.5 h at 37°C (25°C for Pdx1 cultures). IPTG (isopropyl β-D-1 thiogalactopyranoside) was added to the cultures 4 h before harvesting. Cell pellets were lysed in 8 M Urea, 0.1 M NaH_2_PO_4_ at pH 8.0 for 1 h. Cell debris was removed by centrifugation and the cleared lysate was applied to a His-select affinity column (Sigma) pre-equilibrated with lysis buffer. The column was washed with 8 M Urea, 0.1 M NaH_2_PO_4_ at pH 6.3 and proteins were eluted in 8 M Urea, 0.1 M NaH_2_PO_4_ at pH 4.5. Protein fractions were diluted 40× in 20 mM Tris pH 7.6, incubated overnight at 4°C and re-concentrated using a 10 kDa Amicon Centrifugal filter unit (Millipore, Livingston, UK). Final protein concentration was assessed with the Biorad protein assay.

### SDS-PAGE and Western Blotting

Protein aliquots were diluted in NuPage Loading Buffer, run on a 10%, 1 mm, Bis-Tris polyacrylamide gel (both from Invitrogen) and stained with Coomassie Brilliant Blue. Gels used for immunoblotting were transferred to a nitrocellulose membrane, and probed with rabbit anti-Pdx1 antibody [6], 1∶1000 or rabbit anti-MafA antibody, 1∶200 (Santa Cruz Biosciences, Heidelberg, Germany). Subsequently, blots were incubated with a horseradish peroxidase conjugated anti-rabbit IgG antibody (1∶5000).

### RT-qPCR

RNA was extracted using Trizol® reagent (Invitrogen). After digestion with DNase I (Invitrogen) to remove any contaminating DNA, 1 µg of RNA was used for cDNA synthesis. Quantitative Polymerase Chain Reactions (RT-qPCRs) were then performed using the TaqMan gene expression assays (Tables 1 and 2, Applied Biosystems, Paisley, UK). Real-time PCR mixtures were prepared as described by the manufacturer (SensiMiX, Bioline, London, UK) for each gene, denatured at 95°C for 15 seconds and then cycled at 95°C for 30 seconds, 60°C for 30 seconds and 72°C for 10 seconds during 50 cycles, followed by final extension at 72°C for 10 minutes. QPCRs were run in a Roche Lightcycler 480® in triplicate and normalized to GAPDH in the same run. Data was normalized to mouse embryonic stem cells or untreated cells, using the 2^−ΔΔCT^ method [15]. Statistical analysis was performed using One Way ANOVA.

**Table 1:** List of mouse TaqMan Gene Expression Assays used for RT-qPCR.

Gene	TaqMan® Assay ID
Sox17	Mm00488363_m1
Sox7	Mm00776876_m1
Bry T	Mm00436877_m1
Gsc	Mm00650681_g1
Mixl1	Mm00489085_m1
Foxa2	Mm00839704_mH
Insulin 1	Mm01259683_g1
Insulin 2	Mm00731595_gH
Pdx1	Mm00435565_m1
MafA	Mm00845209_s1
Ngn3	Mm00437606_s1
Pax4	Mm01159036_m1
IAPP	Mm00439403_m1
NeuroD	Mm01946604_s1
Nkx6.1	Mm00454962_m1
Glut2	Mm00446224_m1
Somatostatin	Mm00436671_m1
Glucagon	Mm00801714_m1
Albumin	Mm00446224_m1
Gapdh	Mm99999915_g1
Amylase	Mm02342487_g1

**Table 2:** List of human TaqMan Gene Expression Assays used for RT-qPCR.

Gene	TaqMan® Assay ID
Insulin	Hs00355773_m1
Pdx1	Hs00236830_m1
MafA	Hs01651425_s1
Ngn3	Hs00360700_g1
IAPP	Hs00169095_m1
NeuroD	Hs00159598_m1
Nkx6.1	Hs00232355_m1
Glut2	Hs00165775_m1
Somatostatin	Hs00174949_m1
Glucagon	Hs00174967_m1
Gapdh	Hs99999905_m1

### Immunocytochemistry

Cells were washed in Phosphate Buffer Saline (PBS) and fixed in 4% paraformaldehyde (PFA). Permeabilisation was done by incubating the cells in ice cold methanol at −20°C for 10 min followed by washing in PBS. Non-specific interactions were blocked with 10% goat serum in Tris Buffer Saline with 0.01% Triton X-100 (TBST) (Sigma). Cells were stained with rabbit anti-Pdx1 (1∶1000), rabbit anti-MafA (1∶200), (both from Santa Cruz Biosciences), guinea-pig anti-insulin (1∶100, Abcam, Cambridge, UK) or guinea-pig anti C-peptide (1∶50, Abcam) overnight at 4°C. Cells were then probed with goat anti-rabbit AlexaFluor 594 (1∶400) or goat anti-guinea pig Alexa Fluor 488 (1∶400, both from Invitrogen). Cells were mounted in Vectashield with 4′,6-diamidino-2-phenylindole (DAPI, Vector Labs, Peterborough, UK).

### Immunohistochemistry

Embryoid bodies (EBs) were washed in PBS and fixed in 4% PFA overnight at 4°C followed by embedding in 1% agarose prior to paraffin embedding and sectioning. All sections were cut at a 5 µm thickness. For immunofluorescent staining sections were subjected to heat induced antigen retrieval for 15 min using Tris-EDTA Buffer, pH 9.0 and blocked with 10% goat serum in TBST. The samples were then incubated with primary antibodies rabbit anti-Sox7 (1∶100, Abcam) or rabbit anti-Sox17 (1∶100, Santa Cruz Biotechnology), overnight at 4°C. Sections were then probed with anti-rabbit AlexaFluor 488 (1∶400, Invitrogen) for 1 h at room temperature. Slides were mounted in Vectashield with DAPI (Vector Labs).

## Results

### Purification and Uptake of PTD-Transcription Factors

**Figure 1:**
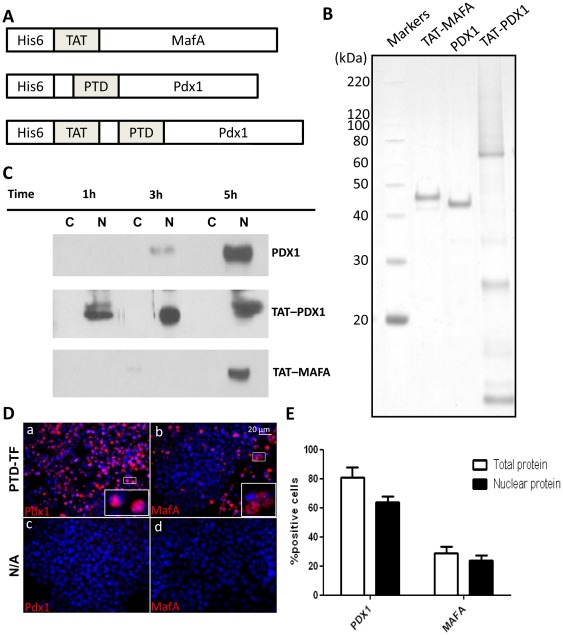
Purification and cellular uptake of TAT-MafA, Pdx1 and TAT-Pdx1. (**A**) Schematic representation of TAT-MafA, Pdx1 and TAT-Pdx1 protein sequences. The PTD box represents the Antennapedia-like protein transduction domain of Pdx1. An histidine tag (His6) was added to the sequence of each protein for binding to the affinity column. (**B**) The purity of recombinant TAT-MafA, Pdx1 and TAT-Pdx1, was assessed on a polyacrylamide gel stained with Coomassie Brilliant Blue. (**C**) Western blot analysis was performed on nuclear (N) and cytoplasmic (C) extracts after incubation of HeLa cells for 1, 3 or 5 hours with 1 µM recombinant Pdx1, TAT-MafA or TAT-Pdx1. (**D**) Immunocytochemistry with anti-Pdx1 and anti-MafA antibodies of mES cells incubated for 4 hours without (N/A) or with (PTD-TF) 1 µM recombinant Pdx1 or TAT-MafA. Inlets of individually stained cells are shown. Nuclei were counterstained with 4′,6-diamidino-2-phenylindole (DAPI). (**E**) Total and nuclear protein uptake was assessed by counting the positive cells from triplicate cultures, where n = 300 cells/replicate. Data are expressed as mean ± SEM.

We have previously shown that mouse and human ES cells can be directed towards an endocrine pancreatic lineage by regulating the expression of the homeodomain transcription factor Pdx1 in response to 4′ hydroxytamoxifen added to the culture media [6]. In the present study we intended to determine whether the activity of Pdx1 could be modulated by adding the transcription factor directly to the cells. In addition, we would extend the study to include the bZIP factor MafA, which plays a role in the late stages of β-cell differentiation [16], [17]. The approach involved generating recombinant transcription factors that were engineered to contain PTD sequences from the HIV1-TAT protein. This was achieved by inserting the cDNAs corresponding to Pdx1 and MafA into pET28b expression vectors, which contain the sequence of the HIV1-TAT protein transduction domain. Given that the Pdx1 sequence already codes for an *Antennapedia* like protein transduction domain [18], the Pdx1 sequence was also cloned into the expression vector pQE-31, which does not contain the TAT sequence (Fig. 1A). These three vectors were then amplified in *Escherichia coli* BL21 (DE3), followed by purification of Pdx1, TAT-Pdx1 and TAT-MafA by histidine affinity chromatography. The purity of the proteins obtained was assessed by SDS-PAGE (Fig. 1B), and the identity of each transcription factor confirmed by Western Blotting and Mass Spectrometry (data not shown). To determine the ability of the purified proteins to translocate into mammalian cells, each protein was added to the culture medium of HeLa cells to a final concentration of 1 µM. Nuclear uptake of the transcription factors was found to be time dependent, with a maximum of nuclear protein detected after addition of the PTD-transcription factors to the cells for a period of 5 hours. No major difference was detected in Pdx1 and TAT-Pdx1 uptake by the HeLa cells after the 5 hour period (Fig. 1C). For this reason, and because TAT-Pdx1 appeared to be subjected to higher amounts of degradation during the purification procedure (Fig. 1B), the subsequent studies were carried out with Pdx1 rather than TAT-Pdx1.

To determine whether Pdx1 and TAT-MafA could be efficiently taken up into mES cells, each protein was added to the mES culture media for a period of 4 hours. After this incubation period, both transcription factors were detected in the cells by immunostaining, with 63.7±11.7% and 23.8±6.1% of cell nuclei positive for Pdx1 and TAT-MafA, respectively. Neither of these transcription factors was detected in untreated mES cells (Fig. 1D, E), indicating that mouse ES cells do not endogenously express Pdx1 or MafA. Collectively these results indicate that recombinant Pdx1 and TAT-MafA are able to directly translocate into the nuclei of mES cells.

### Activin A and BMP-4 Induce Definitive Endoderm formation in mESCs

**Figure 2:**
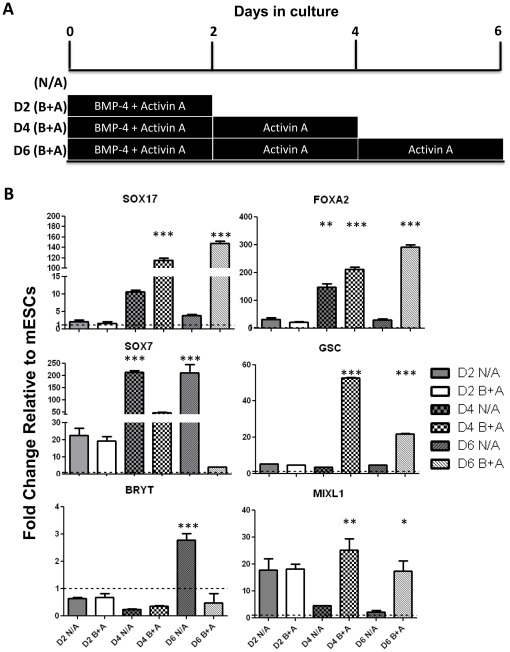
Generation of a Definitive Endoderm enriched population from mouse ES cells. (**A**) Protocol for generation of definitive endoderm (DE) in mouse embryonic stem (mES) cells. Cells were cultured as embryoid bodies in chemically defined medium and treated for 2 days with 100 ng/ml Activin A (A) and 10 ng/ml BMP4 (B), and for the following 2 or 4 days with Activin A alone. (**B**) Treated (B+A) and non treated (N/A) cells were harvested at days 2, 4 and 6 of the differentiation protocol and expression of definitive endoderm (Sox17 and Foxa2), extraembryonic endoderm (Sox7), mesendoderm (Mixl1 and Gsc) and mesoderm (Bry T) markers was determined by RT-qPCR. Data represent the average of triplicate experiments and are expressed as mean ± SEM. Data are expressed as gene expression relative to Gapdh and normalised against day 0 mouse embryonic stem cells (mESCs), where p<0.05 (*), p<0.01(**) or p<0.001 (***).

**Figure 3:**
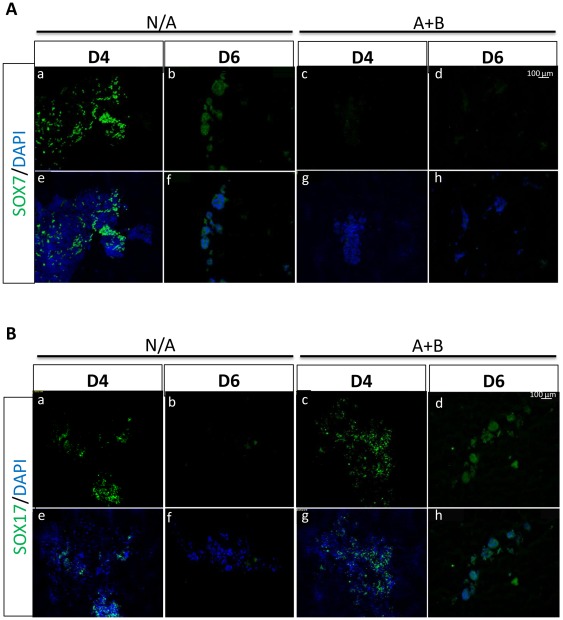
Differentiated DE cells do not express extraembryonic markers. Embryoid Bodies (EBs) were treated with Activin A (A) and BMP4 (B) for a 6 day period. Sections of treated and untreated EBs were made at days 4 and 6 of the differentiation protocol. (**A**) Top panels (a–d) show sections obtained from treated (A+B) and untreated (N/A) EBs stained for the extraembryonic marker Sox7. Bottom Panels (e–i) show counterstaining with the nuclear marker DAPI. (**B**) Top panels (a–d) show sections obtained from treated (A+B) and untreated (N/A) EBs stained for the definitive endoderm marker Sox17. Bottom Panels (e–i) show counterstaining with the nuclear marker DAPI. Data are representative of triplicate experiments.

In the mouse, the pancreas develops from the definitive endoderm [19]. Activin A as a surrogate for Nodal signalling acts as the master inducer of definitive endoderm formation (DE) in culture [3]. It has also been shown that BMP-4 is necessary for promoting DE formation by inducing the formation of the intermediate layer mesendoderm, while Activin A further induces DE differentiation [6]. In this study a similar approach was used to derive DE from mES cells. Undifferentiated mES cells were cultured as embryoid bodies (EBs) for 2 days in chemically defined medium (CDM) containing BMP-4 and Activin A to induce mesendoderm formation. After this period, the EBs were cultured in CDM containing Activin A alone for another 4 days (Fig. 2A). Mesendoderm markers Mixl1 and Gsc were up regulated at day 4 of the differentiation protocol, suggesting that at this stage an intermediary mesendoderm population was being generated. At day 4 the DE markers Sox17 and Foxa2 were already highly up-regulated, indicating that some of the cells had already reached the definitive endoderm differentiation stage. A stronger up-regulation of these endoderm markers was observed at the final stage of the differentiation protocol (day 6), accompanied by the down-regulation of the mesendoderm markers Mixl1 and Gsc, suggesting that more cells in the culture had undergone the transition from mesendoderm to DE (Fig. 2B). This protocol generated an endoderm enriched cell population, which was characterized by the up-regulation of the definitive endoderm markers Sox17 and Foxa2, while the mesoderm marker Brachyury T and the extraembryonic endoderm marker Sox7 were not detected in treated cells (Fig. 2B). These results were confirmed at the protein level by immunocytochemistry, where no Sox7 was detected at either day 4 or day 6 in treated cells, while non treated EBs presented high expression levels of this marker (Fig. 3, top panel). The endoderm marker Sox17 was detected in both non treated and differentiated cells at day 4, however at the end of the differentiation protocol nearly all cells were positive for Sox17 in DE enriched EBs, while non treated cells had lost the expression of the DE marker (Fig. 3, bottom panel). These results indicate that a DE enriched population was efficiently generated after Activin A and BMP-4 treatment.

It was important to determine whether the DE-enriched population of cells would be able to take up the recombinant PTD Transcription Factors (PTD-TFs) from the culture medium. To do this, DE enriched EBs were plated in culture dishes covered with Matrigel™ and allowed to attach for a period of 5 days. On the fifth day of culture the cells were incubated with 1 µM of Pdx1 or TAT-MafA for a period of 4 hours. Staining of the treated cells with specific MafA or Pdx1 antibodies revealed that over 80% of cells were positive for either Pdx1 or MafA (Fig. S1), while non-treated DE-enriched EBs did not express any of the pancreatic transcription factors. These results indicated that the PTD-TFs are also efficiently taken up by ES cells differentiated towards definitive endoderm, thus establishing the conditions necessary for mimicking their expression during pancreatic development.

### PTD-Transcription factors drive DE progenitors towards insulin-producing cells

**Figure 4:**
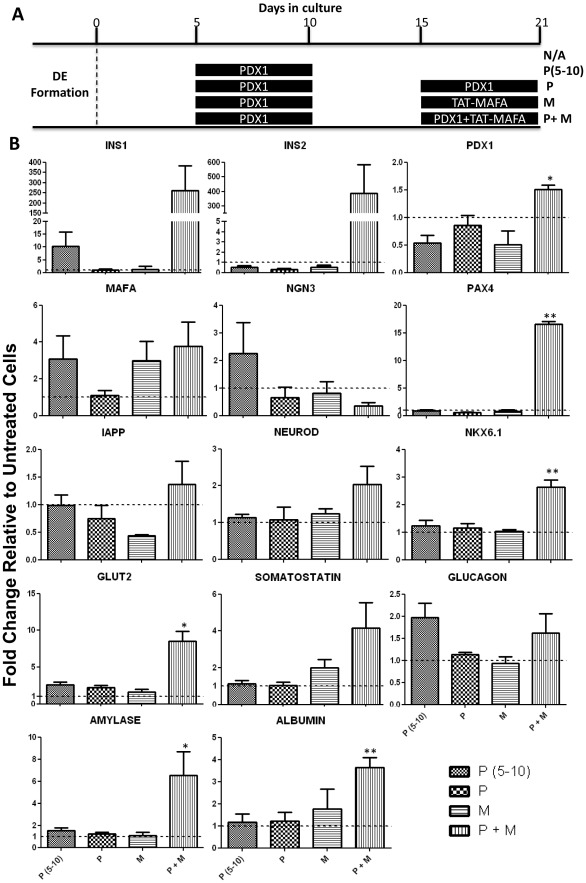
Pdx1 and TAT-MafA promote pancreatic differentiation of DE-enriched mouse ES cells. (**A**) Diagram of the different differentiation culture conditions. After definitive endoderm (DE) formation mES cells were plated as a monolayer and treated for 21 days with the indicated combinations of Pdx1 (P) and TAT-MafA (M). (**B**) On day 21 cells were harvested and total RNA was extracted for analysis of the expression of pancreatic genes by RT-qPCR. Data represent the average of triplicate experiments and are expressed as mean ± SEM. Data are expressed as gene expression relative to GAPDH and normalised against untreated (N/A) cells, where p<0.05 (*), p<0.01 (**) or p<0.001 (***).

To differentiate further the mES cells from DE towards the β-cell phenotype, the recombinant transcription factors Pdx1 and TAT-MafA were administered to the cells in a way that mimics pancreatic development observed *in vivo*. It is known that during pancreatic development Pdx1 is expressed in a biphasic manner, in which the first wave of Pdx1 expression is necessary to specify the pancreatic domain in the primitive foregut [20], [21], while the second wave of Pdx1 expression drives the further differentiation of β-cells [22]. In order to simulate this process *in vitro*, the DE enriched cells were first plated in cell culture dishes and allowed to attach for 5 days. Subsequently, the cells were cultured in the absence or presence of 1 µM Pdx1 for a 5 day period and, again in a second phase, from day 15 to 21 (Fig. 4A). In the second phase of treatment, TAT-MafA was added simultaneously with Pdx1 in order to recapitulate the expression pattern observed during *in vivo* pancreatic development. To determine whether at this later stage Pdx1 or MafA alone would be able to drive the cells towards the β-cell phenotype, these transcription factors were also independently added to the culture (Fig. 4A). After 21 days in culture in the presence of the PTD transcription factors the differentiated cells were harvested and their mRNA levels analysed by RT-qPCR (Fig. 4B). When Pdx1 was added to the culture medium only once, from day 5 to day 10, no pancreatic or liver markers were up-regulated when compared to cells that were not treated with PTD-proteins, suggesting that the first wave of Pdx1 expression was not sufficient to promote formation of β-cells or the pancreatic domain *in vitro*. Similar results were obtained when Pdx1 or TAT-MafA were added independently to the culture during the second expression wave that should drive the formation of β-cells (Fig. 4B). However, when both Pdx1 and TAT-MafA were added simultaneously to the culture, up-regulation of the mouse insulin 1 and 2 genes was observed, as well as the endogenous β-cell transcription factors Pdx1, Nkx6.1, Pax4, NeuroD and the transporter Glut2 (Fig. 4B). Along with the expression of these β-cell markers, other endocrine pancreatic markers were increased, such as the δ-cell marker somatostatin and the exocrine cell marker amylase (Fig. 4B). The fact that the endocrine precursor Ngn3 was decreased in the cells treated with Pdx1 and TAT-MafA is in keeping with the fact that mature beta cells lose the expression of this precursor after differentiation [23]. The liver marker albumin was also up-regulated after this treatment (Fig. 4B), suggesting that a mixed population of cells was obtained, with both liver and pancreatic phenotypes present.

**Figure 5:**
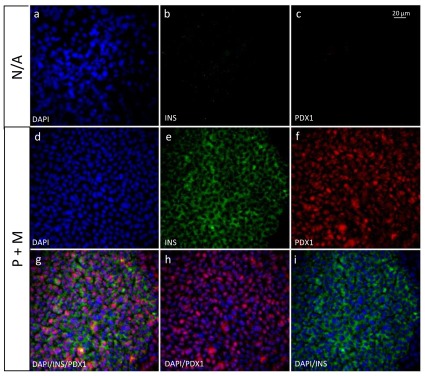
PTD-TF transduced ES cells co-express Pdx1 and insulin. Immunocytochemistry of untreated (N/A) cells or cells treated with Pdx1 from day 5 to 10 and with a combination of Pdx1 and TAT-MafA from day 15 to 21 (P+M). Top panels show staining for the nuclear marker DAPI, Insulin and Pdx1 in untreated cells (a–c). Middle panels show individual staining of DAPI, Insulin and Pdx1 in P+M treated cells (d–f). After treatment with P+M, Pdx1 was detected in the cells nuclei (g, h), while insulin was present in the cytoplasm (g, i). Data are representative of triplicate experiments.

**Figure 6:**
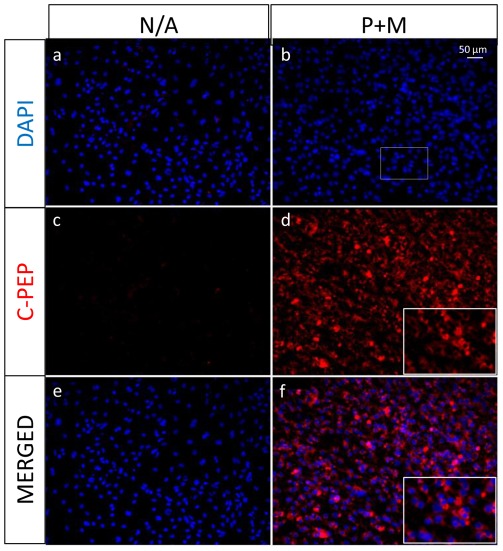
Insulin is synthesized *de novo* by PTD-TF transduced ES cells. After the 21 day differentiation protocol, untreated (N/A) and cells treated with Pdx1 from day 5 to 10 and with a combination of Pdx1 and TAT-MafA from day 15 to 21 (P+M) were stained with a C-Peptide antibody and nuclei counterstained with DAPI. An inlet (b) of a higher magnification is shown in treated samples (d and f). Data are representative of triplicate experiments.

In order to characterize further the phenotype of the cells obtained after the treatment with the combination of PTD-TFs, immunocytochemistry was performed to assess whether the β-cell markers insulin and Pdx1 were present at the protein level (Fig. 5). Double positive insulin and Pdx1 producing cells were detected in cells treated with the combination of Pdx1 and TAT-MafA (P+M cells), while neither insulin nor Pdx1 was detected in cells grown in the absence of the transcription factors. In order to assess whether the detected insulin was being produced *de novo* by the differentiated cells, staining for C-peptide was performed (Fig. 6), confirming that insulin was being produced by the cells and not taken up from the media. C-peptide staining was observed in 83.0%±10.6 (n = 3) of the treated cultures, indicating that the majority of the cells in culture were differentiated towards the β-cell lineage.

**Figure 7:**
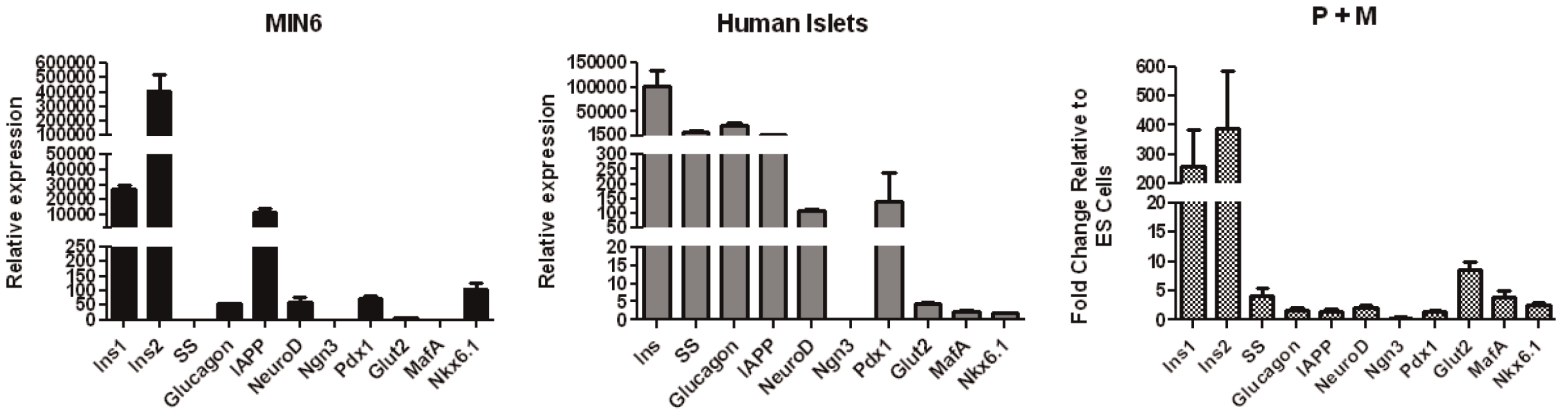
Expression profile of pancreatic markers in PTD-TFs Transduced ES cells, the MIN6 β-cell line and in human islets. Comparison of expression levels between the mouse β-cell line MIN6, human islets and CGR8 cells treated with the combination of Pdx1 and TAT-MafA (P+M). The expression levels of the different markers was analysed by RT-qPCR. Data represent the average of triplicate cultures and are expressed as mean ± SEM relative to GAPDH.

There were a number of interesting features when the pattern of expression of markers in P+M cells was compared with RNA extracted from mouse MIN6 β-cells and human islets (Fig. 7). The MIN6 and P+M cells expressed higher levels of insulin 2 than insulin 1, with roughly equivalent relative levels of Pdx1, Ngn3, NeuroD and Nkx6.1 and relatively higher levels of MafA and the transporter Glut2 in P+M cells. The same was true when P+M cells were compared with human islets, which being a mixed cell population expressed high levels of the somatostatin, glucagon and IAPP. The fact that these were expressed at levels substantially lower than insulin 1 and 2 in P+M cells suggested that the PTD-TF mediated differentiation was preferentially directed towards β-cells. However, the striking observation was the extremely low levels of insulin mRNAs in the P+M cells compare to that in the MIN6 cells or human islets.

## Discussion

Advances in islet transplantation [1], albeit limited [24], have been hindered by a dependence on the availability of cadaveric tissue, and stimulated a drive towards generating a replenishable supply of islets from human pluripotent cells. Despite huge progress in the differentiation of embryonic stem (ES) cells towards pancreas, it has been impossible at this stage to generate a fully functional β-cell *in vitro*, i.e. one that secretes meaningful amounts of insulin in response to glucose in the physiological concentration range [2]. The *in vitro* differentiation of pluripotent cells towards pancreatic lineages is dependent on the addition to the culture media of growth factors and small molecule inhibitors that recreate cell signaling events that occur in the developing embryo. These signaling pathways intersect within the nucleus to establish transcriptional networks that change during the course of development. The importance of these networks was emphasized in our previous study, in which we used a tetracycline-regulatable system to control the activity of an exogenous Pdx1 gene. Thus subtle changes in its temporal expression had a significant effect on the developmental outcome [6]. At present it is possible to generate pancreatic progenitors that can further differentiate into islet-like structures following engraftment in immunocompromised mice [25]. The *in vivo* environment can therefore recapitulate the conditions necessary to establish these networks. We surmised therefore that some of the challenges in generating functional β-cells *in vitro* might be overcome by adding exogenous transcription factors directly to the culture medium.

PTD-TFs have previously been used to study the developing or regenerating pancreas. Thus, administration of a TAT-Ngn3 fusion protein to cultured E9.5 and E13.5 pancreatic explants resulted in efficient uptake and nuclear localisation and an increased level of endocrine differentiation compared to control samples [26], while intraperitoneal injection of recombinant Pdx1 into streptozotocin diabetic mice restored euglycaemia through a combination of β-cell regeneration and liver reprogramming [27]. Here we show that the levels of intracellular Pdx1 can be manipulated by adding Pdx1 protein directly to the culture media. When used in combination with a PTD-MafA protein, we were able to differentiate DE-enriched mES cells towards a population of cells that co-expressed Insulin and endogenous Pdx1, and expressed some of the phenotypic characteristics of a β-cell. The outcome was dependent on adding Pdx1 in two spatially separated phases and the presence of both Pdx1 and PTD-MafA in the second phase.

Although in the present study we were able to detect Insulin mRNA and protein by immunocytochemistry, the levels were below the sensitivity of our C-peptide and insulin assays. Importantly, however when P+M cells were compared with MIN6 β-cells and human islets, although some similarities were found, there were sufficient differences to suggest that additional TFs such as Ngn3, NeuroD and Pax4 that have been shown to play an important role in reprogramming towards islet cells [28], [29] might be required to induce the formation of a more mature β-cell population. Importantly, the levels of expression of insulin mRNA was small when compared to those found in MIN6 cells and human islets. There may be isolated high insulin expressing cells within the P+M population, but this is unlikely since the immunocytochemistry indicated a very even pattern of expression throughout the culture. Although we have shown that the majority of the differentiated ES cells were driven towards insulin producing cells, low mRNA levels of the liver marker albumin were also detected in our cultures. Pancreas and liver share a common endodermal origin and the incomplete differentiation of some of the cells present in our cultures might have resulted in the formation of small numbers of cells of the hepatic lineage.

In conclusion, the present study shows that PTD-TFs can be added to ES cell cultures in a dose and temporal dependent manner. This strategy may provide important insights into the role of TFs in the differentiation of ES cells towards pancreatic lineages, and may inform strategies towards obtaining a more differentiated β-cell in vitro. However, as seen in our related study on the reprogramming of AR42-J exocrine cells [28], PTD-TFs are unlikely to play a major role in generating ES-derived islet cells for therapeutic purposes, because of the low levels of insulin expression obtained when compared with mature β-cells.

## Supporting Information

Figure S1 **PTD-TFs are efficiently taken up by DE-derived ES cells.** (**A**) Immunocytochemistry of definitive endoderm (DE) differentiated cells. Untreated (N/A) cells and cells incubated with Pdx1 or TAT-MafA for a period of 4 hours were stained with antibodies against the DE marker Sox17 and the pancreatic transcription factors Pdx1 or MafA. Top panels show staining for the nuclear marker DAPI, Sox17, Pdx1 and MafA in DE differentiated cells which were not incubated with the PTD-TFs (a–i). Bottom panels show staining for the nuclear marker DAPI, Sox17, Pdx1 and MafA in DE differentiated cells which were incubated with Pdx1 and TAT-MafA for a period of 4 hours (j–r). Data are representative of triplicate experiments. (**B**) Total and nuclear protein uptake was assessed by counting the positive cells from triplicate cultures, where n = 300 cells/replicate. Data are expressed as mean ± SEM.
(TIF)
