# Supplementary figures and images for: Pancreatic Transcription Factors Containing Protein Transduction Domains Drive Mouse Embryonic Stem Cells towards Endocrine Pancreas
Source: PLoS One. 2012 May 1;7(5):e36481. doi: 10.1371/journal.pone.0036481 (PMC3341374; doi:10.1371/journal.pone.0036481)

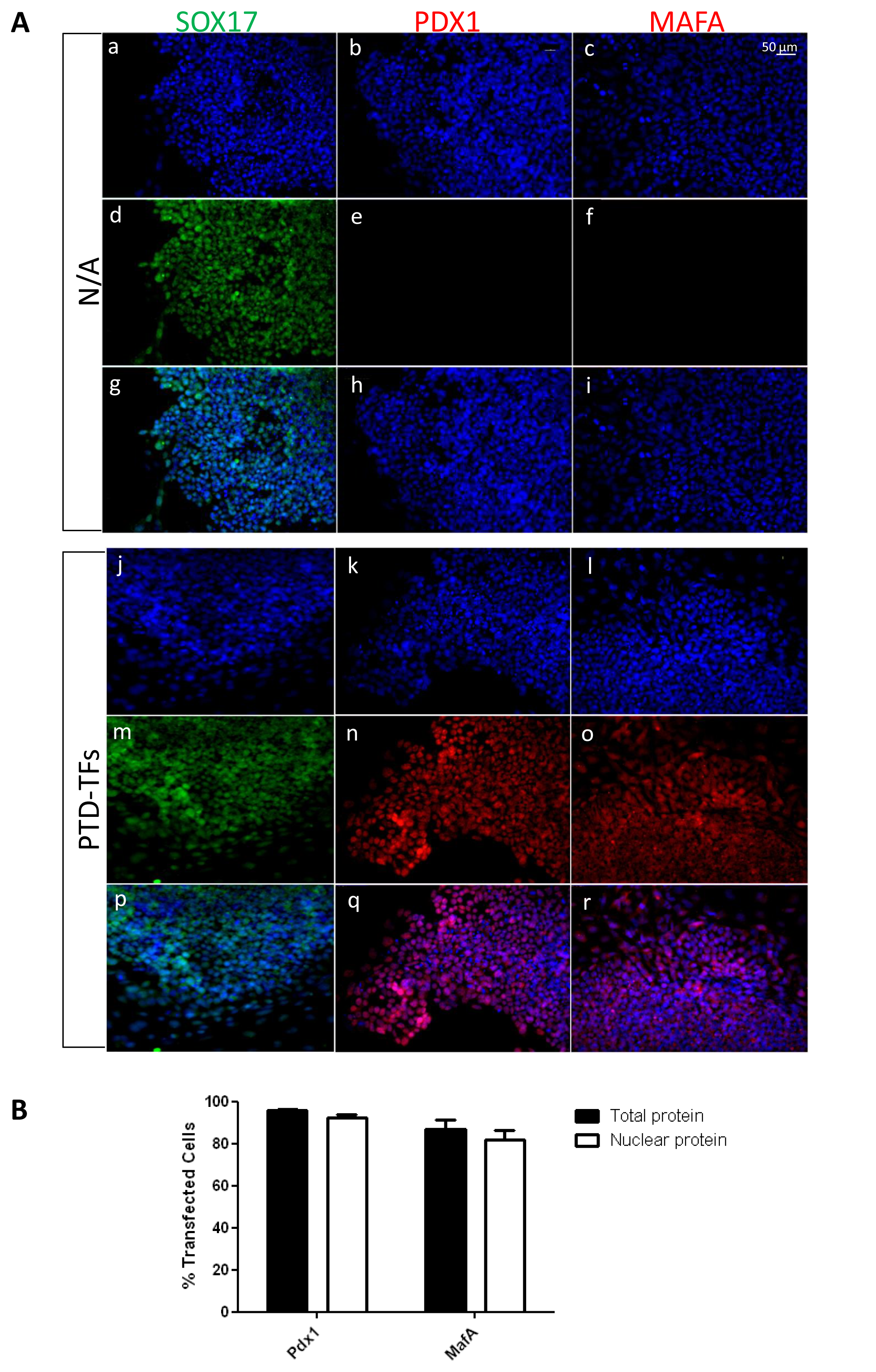

Supplement: Figure S1 — PTD-TFs are efficiently taken up by DE-derived ES cells. (A) Immunocytochemistry of definitive endoderm (DE) differentiated cells. Untreated (N/A) cells and cells incubated with Pdx1 or TAT-MafA for a period of 4 hours were stained with antibodies against the DE marker Sox17 and the pancreatic transcription factors Pdx1 or MafA. Top panels show staining for the nuclear marker DAPI, Sox17, Pdx1 and MafA in DE differentiated cells which were not incubated with the PTD-TFs (a–i). Bottom panels show staining for the nuclear marker DAPI, Sox17, Pdx1 and MafA in DE differentiated cells which were incubated with Pdx1 and TAT-MafA for a period of 4 hours (j–r). Data are representative of triplicate experiments. (B) Total and nuclear protein uptake was assessed by counting the positive cells from triplicate cultures, where n = 300 cells/replicate. Data are expressed as mean ± SEM. (TIF) [file pone.0036481.s001.tif]
